# The micronutrient content in underutilized crops: the *Lupinus mutabilis* sweet case

**DOI:** 10.1038/s41598-022-19202-8

**Published:** 2022-09-07

**Authors:** Miguel Vera-Vega, Jorge Jimenez-Davalos, Gaston Zolla

**Affiliations:** 1 Programa Doctoral en Ciencias e Ingenieria Biologicas, Escuela de Posgrado, Universidad Nacional Agraria La Molina, Lima, Peru; 2 Laboratorio de Fisiologia Molecular de Plantas del PIPS de Cereales y Granos Nativos, Facultad de Agronomia, Universidad Nacional Agraria La Molina, Lima, Peru; 3 Grupo de Investigacion en Mutaciones & Biotecnologia Vegetal, Facultad de Agronomia, Universidad Nacional Agraria La Molina, Lima, Peru

**Keywords:** Biodiversity, Plant sciences, Natural variation in plants, Plant breeding, Plant domestication

## Abstract

Adequate intake of micronutrients is necessary to reduce widespread health issues linked to low intake of iron (Fe), zinc (Zn), boron (B), copper (Cu), and manganese (Mn). Because more than two billion people suffer from micronutrient deficiency globally, to address this problem, highly-nutritious ancestral Peruvian crops like tarwi can be an important component of food security. Thus, our work explores the tarwi micronutrient variability to select biofortified genotypes without affecting seed size and weight. Tarwi is a biofortified food because of its seeds' Fe, Zn, and B content. Furthermore, Boron showed a positive correlation between seed size and weight. At the same time, copper showed a negative correlation. Finally, six accessions (P14, P16, P21, T05, T08, and T25) that are biofortified for Fe, Zn, and B with excellent seed size and weight and with adequate levels of Cu and Mn; adding value to Peruvian biodiversity at a low cost is a starting point for a breeding program to prevent micronutrient disorders.

## Introduction

Tarwi *(Lupinus mutabilis* Sweet) is a legume cultivated in South American Andes, distributed from 2500 to 4500 m above sea level^1^. Its domestication occurred between 1800 and 3450 BP, and a cultivated area of 10,000 ha was estimated in Peru ca. 450 BP^2^. During the colonial and republican times, its consumption decreased and was used only by indigenous communities, maintaining genetic variability until today^3^.

In recent years, tarwi has positioned itself as a superfood because of its high protein and fatty acid content, comparable to soybean^4^, which could make it an excellent replacement for animal protein^5,6^. Unlike other legumes, tarwi is starchless, which is beneficial in reducing the risk of obesity, diabetes, and cardiovascular disease^7^. Medicinally, tarwi contains quinolizine alkaloids, which are toxic secondary metabolites^8^. However, quinolizine alkaloids have antileishmanial and antitrypanosomal properties^9^, whereas sparteine showed in vitro antimicrobial activity against *Mycobacterium phlei*^10^ and *Mycobacterium tuberculosis*^11^. On the other hand, mature tarwi seeds contain conglutin gamma peptides that were able to increase glucose uptake in patients with type 2 diabetes^12,13^. Due to these characteristics, the Peruvian government, through Promperu, pointed out that Peruvian Andean grains such as Tarwi have a tremendous potential demand among the APEC economies. In addition, tarwi is a priority in Bolivia, Ecuador, and Chile, while Argentina and Colombia have a medium priority^14^.

On the other hand, Antúnez de Mayolo^15^ reported that the diet during the Inca empire was balanced. However, changes in eating habits caused during the colonial and republican times and the lack of foresight of the Peruvian government in food security and nutrition have directly affected the nutritional level of children under five years. According to FAO^16^, in 1996, the highest prevalence of chronic malnutrition in children under five years was found in rural areas (40.4%), in the sierra (37.8%), in the jungle (33.0%), and in the departments of Huancavelica (50.3%), Pasco (47.2%), Apurímac (46.9%), Ayacucho (43.2%) and Cusco (40.9%). In 2000, Peru reached levels of chronic malnutrition of 33% in children under five years^17^. Currently, in Peru, 40.1% of children from 6 to 35 months suffer from anemia; almost 700 thousand children under three years of age are anemics out of 1.6 million nationwide^18^. In this sense, anemia in Peru continues to be a public health problem, mainly affecting children and pregnant women. WHO^19^ estimates that 42% of children under five and 40% of pregnant women are anemics.

Additionally, the most common micronutrient deficiencies among women and children are associated with deficiencies in calcium, iodine, iron, selenium, and zinc^20^. Micronutrient deficiency malnutrition is a global problem affecting more than two billion people. Developing countries are the most affected because of the low quality of their diet, characterized by high consumption of carbohydrates but low consumption of vegetables, fruits, animal products, and fish products, which are rich sources of minerals^21^. Traditional strategies to deliver these nutrients have relied primarily on mineral supplementation, dietary diversification, and food fortification^22^. An alternative solution to mineral malnutrition is biofortification, which has been defined as increasing the concentration and/or bioavailability of essential elements in the edible portions of crop plants through agronomic intervention or genetic selection^23–25^. It can be used mainly in developing countries to give extra value to its biodiversity at a low cost.

In this sense, highly-nutritious ancestral Peruvian crops like tarwi could play a significant role in food systems to address food security and reach hunger Zero. Nevertheless, the information on Peru's micronutrient composition of tarwi Genbank is scarce. Thus, the corresponding hypothesis evaluated the positive correlation between micronutrient content and tarwi seed traits (seed length, width, and weight of 100 seeds). The objectives of this study were to evaluate 45 tarwi accessions (1) to determine the iron (Fe), zinc (Zn), Boron (B), copper (Cu), and manganese (Mn) seed content; (2) to measure the phenotypic variations in seed length, seed width, and weight of 100 seeds and (3) to assess the correlation between micronutrient content with seed length, seed width, and weight of 100 seeds.

## Results and discussions

### Micronutrient profile of Lupinus mutabilis

**Figure 1:**
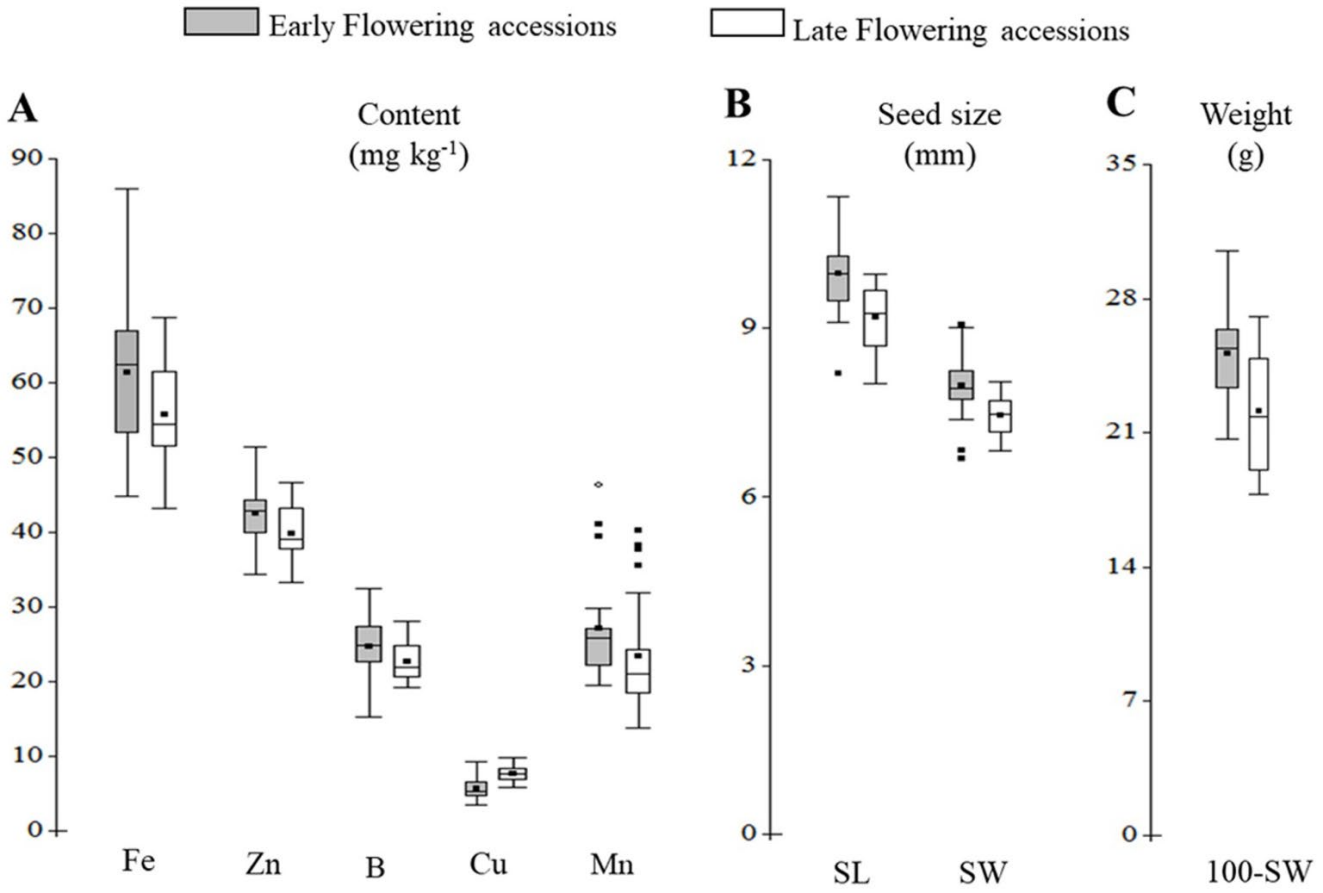
Natural variation in micronutrient content and seed morphological traits among 45 accessions of *L. mutabilis*. (**A**) micronutrient (Fe, Zn, B, Cu, and Mn) content in mg kg^-1^, (**B**) seed length (SL) and seed width (SW), (**C**) 100-Seed Weight (100-SW).

It is critical to have high variability among micronutrients to identify biofortified genotypes for Fe, Zn^26^, B^27^, Cu, and Mn^28^. Thus, Fig. 1A shows greater Fe, Zn, B, Cu, and Mn variability in *L. mutabilis*. Moreover, there was a higher Fe, B, Cu, and Mn content variability in the early-flowering accessions, while Zn variability was higher in late-flowering genotypes (Fig. 1A and Table S2). Additionally, there were statistically significant differences in the accessions' micronutrient concentrations of Fe, Zn, B, Cu, and Mn (Tables 1 and 2). For early-flowering accessions, the Fe concentration was between 46.67 and 88 mg kg^−1^, whereas the range for late-flowering accessions was 44.67–70.33 mg kg^−1^. Without employing a plant breeding strategy, the seed iron concentration in *L. mutabilis* exceeds 40 mg kg^−1^ of Fe^29^; it can be considered a biofortified food. The highest Fe concentration observed in genotypes P03 (Table 1) and T05 (Table 2) was higher than that reported by Sanca^30^, Rodriguez^31^, and Villacres et al.^32^. In contrast, the P03 accession was only higher than Villacres et al.^33^. However, the iron concentrations reported by Ortega-David et al.^34^ were superior to this study.

**Table 1:** Genotype by trait data for 20 early-flowering accessions of *L. mutabilis* for eight traits. Data are expressed as mean ± SD. Significant differences between treatments (*P* < 0.05) are indicated by different letters (ns: no significant differences; *p < 0.05; ***p < 0.001 and ****p < 0.0001).

Accessions code	Micronutrient content (mg kg^-1^)					Size (mm)		Weight (g)
	Fe	Zn	B	Cu	Mn	Length	Width	100-SW
P01	66.33 ± 9^bcdef^	45.33 ± 4^abcde^	25.33 ± 6^ab^	5.67 ± 3^ef^	20.67 ± 2^c^	10.13 ± 0.32^efg^	8.17 ± 0.08^ef^	27.84 ± 0.11^b^
P02	56.00 ± 11^cdefg^	44.00 ± 4^abcde^	24.00 ± 9 ^ab^	6.33 ± 2^cdef^	25.67 ± 4^bc^	10.07 ± 0.20^ fg^	7.72 ± 0.05^gh^	25.33 ± 0.13^def^
P03	88.00 ± 10^a^	48.67 ± 6^abc^	16.67 ± 6^b^	10.67 ± 2^a^	31.00 ± 1^b^	8.49 ± 0.26^i^	7.10 ± 0.08^i^	20.80 ± 0.9^i^
P05	70.67 ± 18^bcd^	44.67 ± 3^abcde^	21.00 ± 8 ^ab^	6.00 ± 2^def^	28.33 ± 2^bc^	9.39 ± 0.08^ h^	6.97 ± 0.13^i^	21.05 ± 0.38^hi^
P06	72.00 ± 6^bc^	43.67 ± 7^abcde^	20.00 ± 9 ^ab^	8.67 ± 0^abcd^	31.00 ± 6^b^	9.52 ± 0.37^ h^	7.66 ± 0.22^ h^	24.5 ± 0.71^ fg^
P07	46.67 ± 6^ g^	52.67 ± 16^a^	25.67 ± 7 ^ab^	10.00 ± 1^ab^	40.67 ± 2^a^	10.42 ± 0.41^def^	8.87 ± 0.20^b^	23.41 ± 0.35^ g^
P08	69.00 ± 4^bcde^	45.67 ± 3^abcde^	29.00 ± 9 ^ab^	6.67 ± 0^cdef^	27.33 ± 12^bc^	10.24 ± 0.19^efg^	8.46 ± 0.12^cde^	25.97 ± 0.95^cde^
P09	46.67 ± 6^ g^	42.33 ± 4^abcde^	24.00 ± 10 ^ab^	8.00 ± 3^abcde^	47.67 ± 7^a^	10.01 ± 0.45^ fg^	8.19 ± 0.24^ef^	22.07 ± 1.22^ h^
P10	65.33 ± 5^bcdef^	47.67 ± 2^abcd^	22.33 ± 6 ^ab^	7.67 ± 0^bcdef^	28.00 ± 5^bc^	10.59 ± 0.16^cde^	8.53 ± 0.14^ cd^	26.52 ± 1.32^ cd^
P11	64.00 ± 3^bcdef^	36.67 ± 1^de^	29.33 ± 2 ^ab^	7.00 ± 1^cdef^	28.33 ± 2^bc^	10.28 ± 0.05^ef^	8.05 ± 0.09f.	25.67 ± 0.84^ef^
P12	54.00 ± 3^efg^	41.00 ± 5^bcde^	34.00 ± 3^a^	9.00 ± 3^abc^	26.67 ± 3^bc^	9.77 ± 0.26^gh^	8.07 ± 0.23f.	25.74 ± 0.15^cde^
P13	52.00 ± 7^ fg^	44.33 ± 2^abcde^	24.00 ± 5 ^ab^	6.00 ± 1^def^	22.00 ± 1^c^	10.78 ± 0.19^ cd^	8.22 ± 0.05^def^	26.71 ± 0.24^c^
P14	64.67 ± 3^bcdef^	40.67 ± 5^cde^	29.33 ± 4 ^ab^	8.33 ± 0^abcde^	24.67 ± 1^bc^	11.43 ± 0.34^ab^	9.29 ± 0.24^a^	28.04 ± 0.78^b^
P16	72.00 ± 5^bc^	35.67 ± 1^e^	31.67 ± 3 ^ab^	7.00 ± 0^cdef^	23.67 ± 1^bc^	11.67 ± 0.19^a^	9.34 ± 0.01^a^	30.64 ± 0.48^a^
P17	61.00 ± 4^bcdefg^	43.00 ± 4^abcde^	28.00 ± 12 ^ab^	6.00 ± 1^def^	23.00 ± 3^bc^	9.49 ± 0.02^ h^	8.09 ± 0.14f.	21.99 ± 0.32^ h^
P18	60.67 ± 6^bcdefg^	46.33 ± 4^abcde^	28.33 ± 9 ^ab^	5.67 ± 1^ef^	27.67 ± 2^bc^	11.05 ± 0.21^bc^	8.85 ± 0.28^b^	26.52 ± 0.42^ cd^
P20	59.67 ± 16^cdefg^	52.00 ± 12^ab^	27.00 ± 10 ^ab^	5.67 ± 1^ef^	23.33 ± 1^bc^	10.49 ± 0.16^def^	8.42 ± 0.11^de^	26.19 ± 0.52^cde^
P21	76.33 ± 9^ab^	36.33 ± 2^e^	31.33 ± 6 ^ab^	6.33 ± 2^cdef^	25.33 ± 1^bc^	10.5 ± 0.09^def^	8.76 ± 0.20^bc^	28.16 ± 0.72^b^
P22	55.00 ± 6^defg^	41.00 ± 1^bcde^	26.33 ± 9 ^ab^	6.67 ± 1^cdef^	42.33 ± 4^a^	10.6 ± 0.52^cde^	7.98 ± 0.37^ fg^	24.14 ± 0.45^ fg^
P25	65.00 ± 10^bcdef^	44.33 ± 4^abcde^	26.33 ± 13 ^ab^	5.00 ± ^f^	21.67 ± 5^c^	10.25 ± 0.07^efg^	8.49 ± 0.07^cde^	23.50 ± 0.86^ g^
Min	46.67	35.67	16.67	5.00	20.67	8.49	6.67	20.80
Max	88.00	52.67	34.00	10.67	47.67	11.63	9.34	30.64
CV (%)	13.30	12.89	29.4	21	14.7	2.57	2.14	2.97
Pr > F	****	*	ns	***	****	****	****	****

**Table 2:** Genotype by trait data for 25 late-flowering accessions of *L. mutabilis* for eight traits. Data are expressed as mean ± SD. Significant differences between treatments (*P* < 0.05) are indicated by different letters (ns: no significant differences; ***p < 0.001 and ****p < 0.0001).

Accessions code	Micronutrient content (mg kg^-1^)					Size (mm)		Weight (g)
	Fe	Zn	B	Cu	Mn	Lenght	Width	100-SW
T01	62.67 ± 11^abc^	37.00 ± 5^ab^	20.33 ± 2^c^	7.00 ± 2^e^	22.33 ± 3^def^	9.14 ± 0.18^ghi^	7.19 ± 0.13^jk^	18.33 ± 0.60^n^
T02	56.33 ± 4^bcde^	39.67 ± 6^ab^	20.67 ± 1^c^	8.00 ± 2^cde^	19.33 ± 2^efgh^	10.20 ± 0.29^ab^	8.18 ± 0.31^abc^	22.36 ± 0.03^i^
T03	53.00 ± 1^cde^	35.33 ± 8^b^	21.33 ± 4^bc^	8.33 ± 1^bcde^	15.00 ± 1^ h^	8.32 ± 0.12^j^	7.08 ± 0.21^ k^	19.24 ± 0.42^ m^
T04	44.67 ± 9^e^	37.00 ± 7^ab^	21.33 ± 4^bc^	9.67 ± 1^abcd^	15.67 ± 3^gh^	8.70 ± 0.32^ij^	7.22 ± 0.14^jk^	18.50 ± 0.29^n^
T05	70.33 ± 8^a^	39.67 ± 5^ab^	23.00 ± 1^abc^	8.00 ± 1^cde^	22.67 ± 2^def^	9.46 ± 0.23^ fg^	7.39 ± 0.21^hijk^	20.94 ± 0.42^j^
T06	63.00 ± 3^abc^	39.00 ± 4^ab^	21.67 ± 3^bc^	9.33 ± 1^abcde^	19.00 ± 2^efgh^	8.96 ± 0.31^hi^	7.52 ± 0.24^fghij^	19.52 ± 0.50^ lm^
T07	61.00 ± 2^abcde^	42.00 ± 4^ab^	23.33 ± 7^abc^	9.00 ± 1^abcde^	20.67 ± 2^defg^	8.66 ± 0.10^ij^	7.48 ± 0.09^ghij^	18.99 ± 0.51^ mn^
T08	52.67 ± 2^cde^	40.33 ± 8^ab^	25.00 ± 1^abc^	10.00 ± 0^abc^	22.67 ± 2^def^	9.78 ± 0.06^abcdef^	7.81 ± 0.11^cdefg^	22.29 ± 0.27^i^
T09	66.00 ± 5^abc^	41.00 ± 8^ab^	22.00 ± 4^bc^	8.00 ± 1^cde^	23.67 ± 5^cde^	10.06 ± 0.08^abcd^	8.14 ± 0.07^abc^	24.64 ± 0.17^efg^
T10	67.33 ± 4^ab^	45.67 ± 4^ab^	21.33 ± 3^bc^	7.33 ± 1^de^	39.67 ± 5^a^	8.95 ± 0.20^hi^	7.56 ± 0.34^efghij^	19.42 ± 0.16^ lm^
T11	52.67 ± 1^cde^	43.33 ± 10^ab^	23.33 ± 1^abc^	8.00 ± 2^cde^	19.33 ± 2^efgh^	9.70 ± 0.05^cdef^	7.72 ± 0.21^defgh^	25.46 ± 0.16^bcd^
T12	63.33 ± 4^abc^	45.33 ± 13^ab^	28.00 ± 3^ab^	11.00 ± 2^a^	39.00 ± 2^a^	9.82 ± 0.45^abcdef^	8.02 ± 0.39^abcd^	27.61 ± 1.14^a^
T13	57.33 ± 2^abcde^	41.00 ± 7^ab^	26.33 ± 2^abc^	9.67 ± 2^abcd^	41.67 ± 4^a^	9.51 ± 0.27^efg^	7.90 ± 0.30^bcdef^	26.13 ± 0.23^b^
T14	63.33 ± 2^abc^	45.67 ± 8^ab^	25.67 ± 4^abc^	10.00 ± 1^abc^	28.33 ± 3^c^	9.32 ± 0.19^fgh^	7.66 ± 0.40^defghi^	25.12 ± 0.38^def^
T15	55.33 ± 9^bcde^	48.67 ± 4^a^	29.33 ± 4^a^	9.67 ± 1^abcd^	37.00 ± 5^ab^	9.38 ± 0.25^fgh^	7.73 ± 0.28^defgh^	24.12 ± 0.33^ g^
T16	53.67 ± 14^bcde^	46.33 ± 9^ab^	26.33 ± 2^abc^	9.33 ± 1^abcde^	18.33 ± 2^fgh^	8.94 ± 0.20^hi^	7.36 ± 0.11^hijk^	21.65 ± 0.38^i^
T17	48.33 ± 10^de^	45.33 ± 7^ab^	22.67 ± 5^abc^	8.00 ± 1^cde^	21.00 ± 1^def^	9.73 ± 0.06^bcdef^	7.95 ± 0.15^abcde^	26.07 ± 0.58^bc^
T18	56.67 ± 11^bcde^	39.67 ± 3^ab^	26.33 ± 7^abc^	8.33 ± 1^bcde^	19.67 ± 2^efgh^	10.17 ± 0.07^abc^	7.84 ± 0.19^cdefg^	25.43 ± 0.55^bcd^
T19	48.00 ± 9^de^	38.00 ± 4^ab^	23.00 ± 5^abc^	7.67 ± 2^cde^	23.67 ± 2^cde^	9.97 ± 0.16^abcde^	7.92 ± 0.27^abcde^	27.18 ± 0.21^a^
T20	56.00 ± 8^bcde^	42.67 ± 5^ab^	21.67 ± 4^bc^	9.00 ± 2^abcde^	33.33 ± 4^b^	9.57 ± 0.17^efg^	7.28 ± 0.33^ijk^	18.31 ± 0.25^n^
T21	53.67 ± 3^bcde^	38.00 ± 3^ab^	21.33 ± 6^bc^	9.33 ± 1^abcde^	20.33 ± 1^efg^	8.91 ± 0.11^hi^	7.41 ± 0.32^hijk^	20.03 ± 0.39^kl^
T22	54.67 ± 8^bcde^	40.00 ± 7^ab^	22.33 ± 3^abc^	7.00 ± 1^e^	25.67 ± 2^ cd^	10.19 ± 0.38^abc^	8.32 ± 0.43^a^	25.35 ± 0.06^cde^
T23	55.33 ± 13^bcde^	48.00 ± 7^ab^	26.00 ± 2^abc^	10.67 ± 1^ab^	21.33 ± 1^def^	10.17 ± 0.09^abc^	8.26 ± 0.07^ab^	24.45 ± 0.47^ fg^
T24	67.00 ± 3^ab^	44.33 ± 5^ab^	25.67 ± 1^abc^	9.33 ± 1^abcde^	21.00 ± 1^def^	9.60 ± 0.14^defg^	8.00 ± 0.19^abcd^	23.37 ± 0.24^ h^
T25	47.00 ± 5^e^	43.67 ± 5^ab^	26.33 ± 2^abc^	9.00 ± 0^abcde^	25.67 ± 1^ cd^	10.27 ± 0.09^a^	7.97 ± 0.28^abcd^	20.52 ± 0.26^jk^
Min	44.67	35.33	20.33	7.00	15.00	8.32	7.08	18.31
Max	70.33	48.67	29.33	11.00	41.67	10.27	8.32	27.61
CV (%)	12.27	15.69	14.89	13.97	10.78	2.71	2.72	1.85
Pr > F	***	ns	ns	***	****	****	****	****

The Zn concentration in early-flowering accessions was between 35.67 and 52.67 mg kg^−1^ and for late-flowering accessions between 35.33 and 48.67 mg kg^−1^, being considered as Zn-biofortified^29,35^ as well. P07 and T15 genotypes showed the highest Zn concentrations (Tables 1 and 2). The Zn content reported in *L. mutabilis* by Villacres et al.^33^, Rodriguez^31^, and Ortega-David et al.^34^ were lower than those of P07 and T15. However, the higher Zn concentrations over P07 and T15 were reported by Sanca^30^ and Villacres et al.^32^.

The high levels of Fe and Zn observed in tarwi (Fig. 1A) could be explained by Zhao et al.^36^ work. He suggests a possible link between grain protein and the levels of the two trace elements under the control of a NAC transcription factor (NAM-B1) that accelerates senescence and increases the remobilization of nutrients (N, Fe, and Zn) from leaves to developing grains^37^. Though Zhao's findings need to be validated in tarwi, they are relevant to this study because the tarwi seed protein content is higher than 40%^6^.

Due to massive reports on B-deficiency in cropping systems^38^, biofortification is a sustainable option to increase the yield and quality of crops^39^. Boron levels in food are critical because it is involved in the formation and hardness of bone structure^40^. Under this consideration, Boron concentration in the late and early-flowering accessions was between 20.33 and 29.33 mg kg^−1^ and 16.67 and 34 mg kg^−1^, respectively (Tables 1 and 2), where T15 and P12 are unique genotypes to generate B biofortified cultivars. Although the T15 genotype showed high levels of B and Zn, its iron level was 55.33 mg kg^−1^. Since the interaction between B and Zn affects Fe transport and Fe content in organs^41^.

Despite the significant variability in Cu content, genetic selection should focus on a balanced intake of this micronutrient (≤ 10 mg kg^−1^) because consuming foods rich in Cu may increase the incidence of neurodegenerative pathologies like Alzheimer's^42^. According to Schilsky^43^, the adequate concentration of Cu within a balanced diet should not be higher than 11 mg kg^−1^. Thus, Cu concentration in the late and early-flowering groups of tarwi is between 7 and 11 mg kg^−1^ and 5 and 10.67 mg kg^−1^, respectively (Table 1 and 2). According to the Schilsky^43^ criterion, T08, T12, T14, P03, and P07 genotypes have an adequate Cu concentration for a balanced diet, unlike tarwi genotypes studied by Villacres et al.^33^, Rodriguez^31^ and Ortega-David et al.^34^.

Like Cu, a high intake of manganese can cause neurodegenerative problems^44^. According to Falah et al.^45^, the Mn content in a balanced diet should contain 18 mg kg^−1^ for women and 22 mg kg^−1^ for men. Tarwi Mn content was between 20.67 and 47.67 mg kg^−1^ (Tables 1 and 2), where the late-flowering genotypes T03 and T04 presented the lowest Mn concentration (Table 2). However, the highest Mn concentrations were observed in P09 (47.67 mg kg^−1^), P22 (42.33 mg kg^−1^), T10 (39.67 mg kg^−1^), T12 (39 mg kg^−1^), T13 (41.67 mg kg^−1^) and P07 (40.67 mg kg^−1^) accessions, see Table 1 and 2. Similarly, Ortega-David et al.^34^ reported genotypes with higher concentrations of Mn in *L. mutabilis*.

In contrast, Rodriguez^31^ reported accessions with lower amounts of Mn for this species. On the other hand, Villacres et al.^33^ affirm that the Mn content can be reduced by up to 50% during the debittering process of seeds, turning them into safe foods^45^. In this sense, the maximum Mn concentration in seeds without debittering should not exceed 36 mg.kg^−1^ to be included in a balanced diet. Furthermore, more than 90% of absorbed manganese is mainly excreted via the bile into the feces^46^.

### Seed morphological traits

Since crop domestication, the yield has been one of the most important agronomic traits in plant breeding^47^. Yield components include seed traits such as seed size (SL and SW) and 100-SW^48^; these characteristics showed considerable variability in early-flowering accessions than in late-flowering accessions (Fig. 1B,C) and were statistically significant differences for each group (Table 1 and 2). In tarwi, there is a high correlation between 100-SW and yield^49^. The 100-SW ranged from 20.80 to 30.64 g for the early-flowering genotypes (Table 1) and 18.31 to 27.61 g for late-flowering genotypes (Table 2), with the accessions P14, P16, P21, and T12 exhibiting the largest 100-SW. Furthermore, tarwi 100-SW was higher than *Lupinus angustifolius* L., *Lupinus cosentinii* G., *Lupinus digitatus* F., *Lupinus hispanicus* B&R., *Lupinus luteus* L. and *Lupinus micranthus* G.^50^. However, our findings on 100-SW were similar to Aguilar-Angulo^51^ but higher than De La Cruz^52^, Huisa^53^, Cayo^54^, Buircell and Cowling^55^, Plata^56^, Mendoza^57^, Aguilar^58^, Aquino^59^, Atchison et al.^2^ and Barda et al.^60^.

Seed size is an important agronomic trait because of its importance for consumers and industry^61^. Thus the genotypes with large seed sizes (AS and LS) were P14, P16, T02, and T22 (Tables 1 and 2). This pattern was also observed in eight genotypes by Cayo^54^. Additionally, P14 and P16 genotypes displayed excellent seed morphological characteristics (AS, LS, and 100-WS) with a high content of micronutrients (Fig. 1A), demonstrating the crop's excellent potential for food and feed industries.

### Selection of tarwi accessions

**Figure 2:**
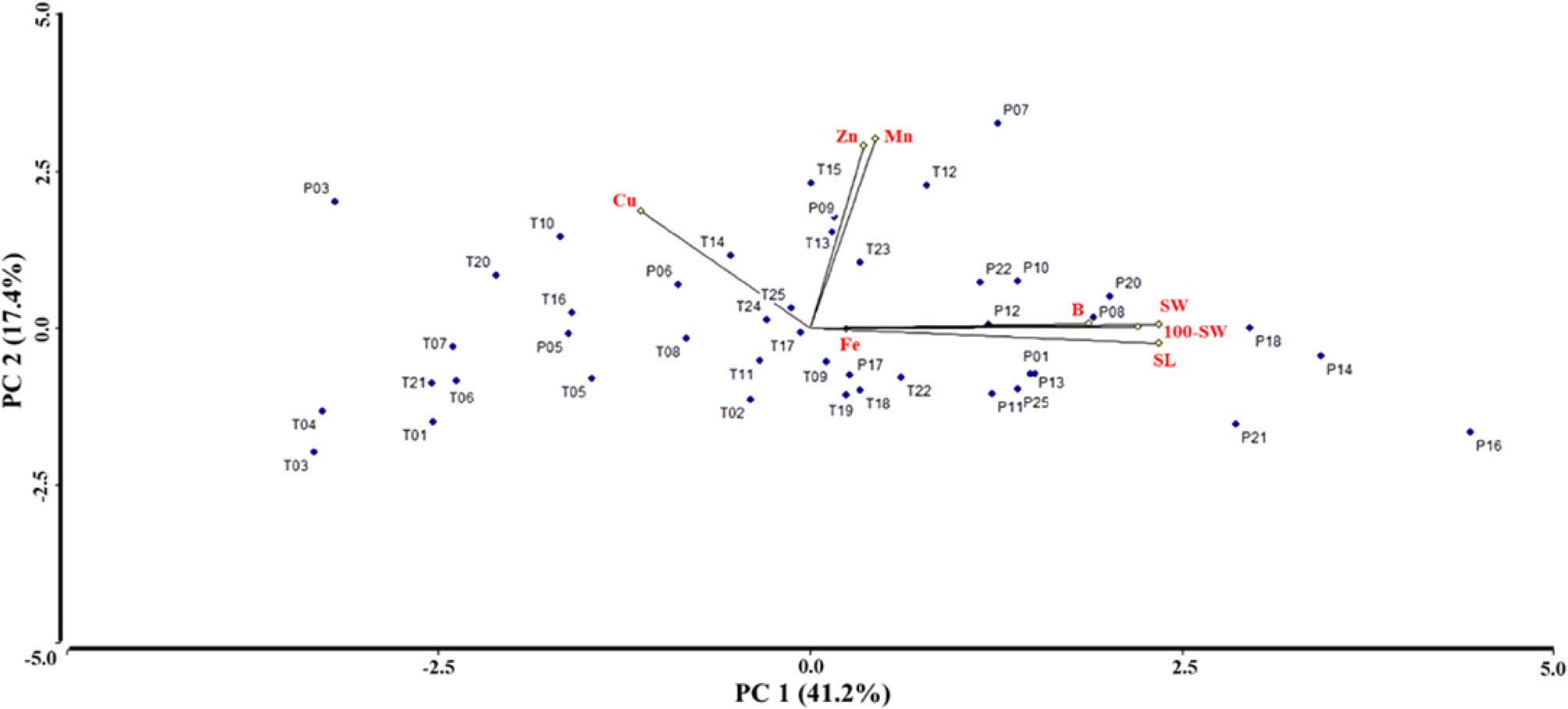
Biplot of principal component analysis (PCA) based on the micronutrient content (Fe, Zn, B, Cu and Mn) and seed morphological traits (SL, SW and 100-SW) for 45 accessions of *L. mutabilis*.

The first three components in the principal component analysis (PCA) account for 72.2% of the total variability (Table 3). The weight of 100 seeds (100-SW), seed length (SL), seed width (SW), and Boron content (B) are related to PC1 (41.2%). The Mn and Zn contents are closely related to PC2 (17.4%), and the iron content (Fe) is closely related to PC3 (13.6%). In PC1, the morphological characteristics showed a high correlation with B (Table 3, Fig. 2, and Table S3), which can be attributed to B's role in seed development and size^62^, which can boost crop yields^63^. The high B concentration in *L. mutabilis* may be due to the high efficiency of B transport to the seeds. Furthermore, B can modulate the absorption and translocation of nutrients to the seed^64^, allowing a higher absorption of Fe, Zn, and Cu^65^ but less in Mn^66^.

**Table 3:** Eigenvectors and eigenvalues of the first three principal components of 8 traits.

Variables	PC1	PC2	PC3
Eigenvalue	3.30	1.39	1.09
Variation (%)	41.2	17.4	13.6
Cumulative (%)	41.2	58.6	72.2
Fe	0.10	−0.01	0.91
Zn	0.14	0.75	0.19
B	0.74	0.02	−0.27
Cu	−0.45	0.48	−0.37
Mn	0.17	0.78	0.05
SL	0.93	−0.06	−0.12
SW	0.93	0.02	0.01
100-SW	0.87	0.00038	0.14

However, the relationship between Cu and seed size was inverse and significant; because, in high concentrations, copper reduces the seed size, impacting grain yield and quality^67^. However, the only two micronutrients with a significant and positive correlation were Mn and Zn (Table S3), and Biplot-PCA corroborates it (Fig. 2). This may show a synergistic effect between these two micronutrients^68^, suggesting a cotransport of Mn and Zn.

**Figure 3:**
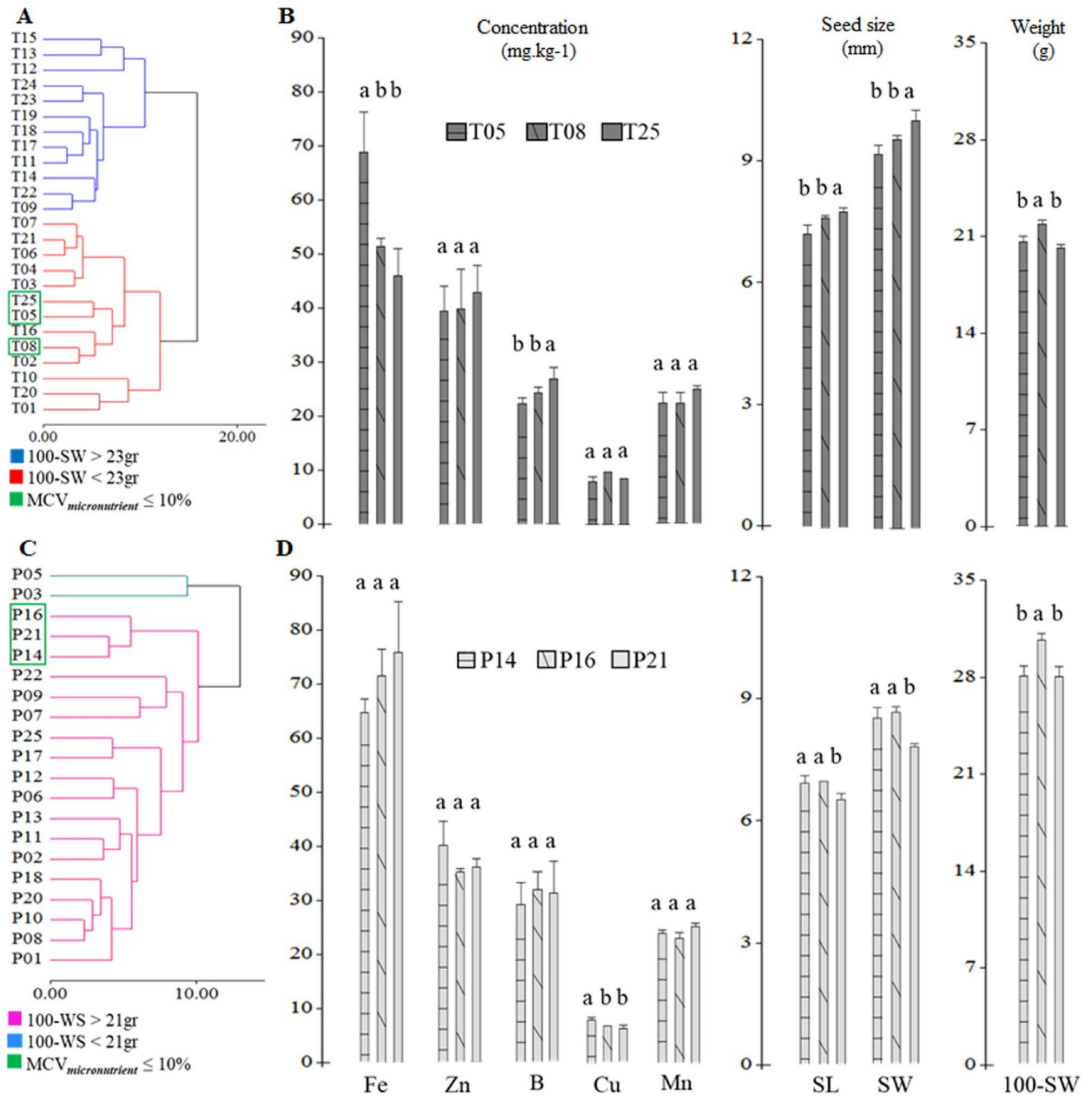
Cluster analysis and selection of late-flowering and early-flowering tarwi genotypes. (**A** and **C**) cluster analysis and selected material according to their micronutrient content (Fe > 40 mg kg^−1^, Zn > 28 mg kg^−1^, B > 13 mg kg^−1^, Cu < 9 mg kg^−1^ and Mn < 30 mg kg^−1^) and mean-coefficient of variation for micronutrient (MCV_*micronutrient*_) ≤ 10% (**B** and **D**) description of the best genotypes.

Since 100-WS is highly associated with yield in *L. mutabilis*^49^ and seed size can affect it, the Mahalanobis cluster analysis was used to select the best genotypes. Thus, the late-flowering accessions were divided into 12 accessions with 100-SW > 23 g and 13 accessions with 100-SW < 23 g (Fig. 3A). Simultaneously, 20 early-flowering accessions were divided into two subgroups, 2 genotypes with 100-SW < 21 g and 18 genotypes with 100-SW > 21 g (Fig. 3C). To narrow down the selection, results based on the micronutrient criteria (Fe > 40 mg kg^−1^, Zn > 28 mg kg^−1^, B > 13 mg kg^−1^, Cu < 9 mg kg^−1^ and Mn < 30 mg kg^−1^) and a mean coefficient of variation of micronutrients (MCV_*micronutrient*_ ≤ 10%) were used and allowed to select three late-flowering accessions (T05, T08 and T25) and three early-flowering accessions (P14, P16 and P21) that were biofortified for Fe, Zn and B with adequate nutritional requirements in Cu and Mn and excellent seed size and weight (Fig. 3C,D and Table S4), unlike than Villacres et al.^33^, Rodríguez^31^ and Ortega-David et al.^34^. Furthermore, a MCV_*micronutrient*_ value of  ≤ 10% among the selected accessions could be due to a high specificity of the proteins associated with the absorption, translocation, remobilization and/or storage of these micronutrients. Therefore, a better understanding of these processes is required to explain the micronutrient homeostasis of tarwi. Moreover, our data demonstrated that the selection of micronutrient-rich tarwi genotypes was achieved without affecting the morphological traits of the seeds (Fig. 3 and Table S4). Finally, its unique micronutrient levels make it a suitable grain to be included in a healthy diet.

## Materials and methods

### Plant material

In 2014, the regeneration of the tarwi Genbank was carried out in the Santa Ana experimental station at INIA-Huancayo. The INIA data on days to flowering was taken when the plot was at 50% of flowering. Thus, 20 early-flowering (86–122 days) and 25 late-flowering (140–166 days) accessions of *Lupinus mutabilis* S. used in this study were given in 2015 to Universidad Nacional Agraria La Molina under a material transfer agreement (ATMG 001–2015). The germplasm used was collected in Cuzco, Cajamarca, Ancash, Junin and Huanuco. Finally, Lupin Descriptors^69^ was used to characterize tarwi seed (Table S1). All the measurements for this study were done between 2019 and 2020 at Universidad Nacional Agraria La Molina.

### Sample preparation

All samples were weighted with a Henkel balance (± 0.01) and dried until constant weight in an oven at 70 °C. Dry samples were ground with a Thomas Model 4 Wiley® Mill (Thomas Scientific) until a homogeneous mass was obtained. All samples were kept in sealed containers to avoid contact with atmospheric humidity.1 g of ground sample was weighed with an OHAUS PA313 balance (± 0.001 g) and used for sample digestion.

### Analysis of the micronutrients profile (Fe, Zn, B, Cu, and Mn) and seed size and weight

In *L. mutabilis* seeds, Fe, Zn, Cu, and Mn concentrations were measured by the wet digestion method^70^. The samples were digested in a nitroperchloric mixture and measured by atomic absorption spectrometry (Perkin Elmer Analyst 200). The curcumin-acetic acid method^72^ determined boron (B) concentration. The micronutrient concentration was expressed in mg kg^−1^.

However, seed length (SL), seed width (SW), and the weight of 100 seeds (100-SW) were determined according to Pereira et al.^71^. Seed size was expressed in millimeters (mm), and the weight of 100 seeds in grams (g). All experiments were done in triplicate. The Pearson correlation analysis was performed in Excel to determine the relationship between variables. Finally, boxplots, bar-graphs, mean, standard deviation, and coefficient of variation (CV %) were calculated using the statistical InfoStat analysis system (www.infostat.com.ar) software. Duncan’s multiple range test was used to separate the differences in the mean scores at a significance level of *P* < 0.05.

### Selection of tarwi accessions

The data in early and late-flowering genotypes of *L. mutabilis* (Table S2) were standardized to estimate the genetic distance matrix using the Euclidean distance^72^. The analysis was carried out using InfoStat software (www.infostat.com.ar), and PCA and Biplot of PCA consistency were verified by cophenetic correlation coefficient^73^.

To identify the best traits, the cluster analysis was done first, where Mahalanobis distance was used as the distance matrix^74^ with InfoStat software (www.infostat.com.ar); and to narrow the selection results based on healthy eating, the micronutrient criteria: Fe > 40 mg kg^−1^ ^29^, Zn > 28 mg kg^−1^ ^29^, B > 13 mg kg^−1^ ^75^, Cu < 9 mg kg^−1^ ^76^, and Mn < 30 mg kg^−1^ ^77^ and mean-coefficient of variation for micronutrient (MCV_*micronutrient*_) of less or equal than 10%^78^ were used.

## Conclusions

Without employing a plant breeding strategy, Fe, Zn, and B content in tarwi seeds exceed the 40 mg kg^−1^, 28 mg kg^−1^, and 13 mg kg^−1^, respectively, and it can be considered biofortified food. Moreover, Boron stands out as an important micronutrient because of its positive correlation with seed size and seed weight because B is involved in the development and size of the seeds. However, Cu and seed size showed a negative correlation, helping the selection of genotypes with good seed morphological traits and adequate Cu concentration (≤ 10 mg kg^−1^). These results do provide partial validation of the hypothesis.

100-WS, along with micronutrient criteria for healthy eating and MCV_*micronutrient*_ ≤ 10% allowed to select six accessions (P14, P16, P21, T05, T08, and T25) that were biofortified for Fe, Zn and B with excellent seed size and weight and adequate levels of Cu and Mn. These genotypes will serve as the basis for a breeding program to boost micronutrient content, which will help avoid micronutrient problems and reduce healthcare expenditures by promoting healthy eating. Lastly, fostering well-being and ensuring healthy eating/living is crucial for sustainable development. Biofortification has a crucial role in reducing health costs by promoting healthy eating. However, it has limitations because the technology does not address the high-cost analysis to evaluate large seed banks in megadiverse countries. Thus, other technologies such as Micro-XRF and benchtop TXRF spectrometers could be an alternative to reduce analysis costs and build a high-throughput mineral-nutrient phenotyping platform.

## Supplementary Information

Supplementary Information 1.

Supplementary Information 2.

Supplementary Information 3.

Supplementary Information 4.

## Data Availability

All relevant data are within the paper and its supporting Information files.
